# Editorial: Next generation γδ T cell-based tumor immunotherapy

**DOI:** 10.3389/fimmu.2022.1041362

**Published:** 2022-09-30

**Authors:** Andy Hee-Meng Tan, Alice Man Sze Cheung, Dieter Kabelitz

**Affiliations:** ^1^ Immune Cell Manufacturing, Bioprocessing Technology Institute Agency for Science, Technology and Research (ASTAR), Singapore, Singapore; ^2^ Department of Hematology, Singapore General Hospital, Singapore, Singapore; ^3^ SingHealth Duke-National University of Singapore (NUS) Medicine Academic Clinical Programme, Singapore, Singapore; ^4^ Institute of Immunology, University of Kiel, Kiel, Germany

**Keywords:** gamma delta (γδ) T cells, tumor immunotherapy, butyrophilins, cytokine programming, gamma delta TCR repertoire, cost-effective manufacturing of gamma delta t cell therapies, chimeric antigen receptor-engineered gamma delta T cells, phosphoantigens

The discovery of the γδ T-cell receptor (TCR) and its ability to confer potent cytotoxic activity in CD3+ cells some 35 years ago sparked the initial proliferation in research that garnered widespread interest in the biology and function of γδ T cells. The identification of major human γδ TCR clonotypes, their tissue distributions as well as dynamic changes throughout ontogeny and in disease states have contributed to the appreciation of human γδ T cell diversity. Despite this, incomplete understanding of mechanisms underlying the complexity of various γδ T cell subsets in homeostasis, inflammation and malignancy restricted the focus of most early studies to blood circulating Vγ9Vδ2 T cells which could be robustly activated and expanded *ex vivo* using aminobisphosphonates such as zoledronate compared with other γδ T cell subsets for which activating ligands were then largely unknown. This galvanized attempts to harness the tumoricidal potential of Vγ9Vδ2 T cells in clinical trials. Although these cells exhibited highly promising safety profiles in patients, early trial data revealed suboptimal anti-tumor efficacy. Despite these setbacks, recent breakthroughs in deciphering the unique antigen (Ag) binding modes of γδ TCR coupled with high dimensional analyses of tissue- and disease-specific γδ T cell subsets at single cell resolution led to renewed excitement in the development of γδ T cell-based therapeutics. This Research Topic has compiled a series of nine articles of which five review our hitherto understanding of the multifaceted nature of γδ T cells and four report original research providing new insights into the molecular and cellular regulation of a diverse repertoire of γδ T cells, paving the way for next generation γδ T cell-based tumor immunotherapies.

Despite extensive use of phosphoantigens (pAgs) to activate and expand Vγ9+Vδ2+ T cells, the role of butyrophilins (BTNs) in mediating Vγ9Vδ2 TCR-dependent activation is only gaining recent appreciation. Expression of BTN3A1 and BTN2A1 heterodimers, in complex with pAgs, on the surface of accessory innate immune, infected or tumor cells is an absolute requirement for interaction with the Vγ9Vδ2 TCR. Other BTN and BTN-like (BTNL) molecules are involved in the ontogeny and homeostasis of non-Vγ9+Vδ2+ T cell subtypes as noted by Herrmann and Karunakaran. Their review also emphasized dissection of BTN-associated mechanisms that enhance the effector function to broaden the therapeutic applications of γδ T cells in disease settings. Extending the role of TCRδ complementarity-determining region 3 (CDR3δ) in Vγ9Vδ2 TCR-mediated responses, Vyborova et al. adduced evidence for CDR3δ determinants in pAg sensing that are significantly correlated with Vγ9Vδ2 TCR affinity and signal strength, contributing to Vγ9Vδ2 T cell repertoire focusing. Furthermore, surface expression of the inhibitory natural killer receptor (NKR) CD94/NKG2A is biased toward while that of activating NKR NKG2D is independent of these CDR3δ traits, findings which may impact design of high-affinity Vγ9Vδ2 TCR-based therapies.

γδ T cells manifest substantial plasticity and can be polarised to different cell states in response to environmental stimuli. It is thus not uncommon to identify γδ T cells with opposing functional properties in different biological contexts. The mini review by Bhat et al. provides a comprehensive summary of studies highlighting the dichotomous nature of γδ T cells influenced by diverse pathological milieux, leading to beneficial or detrimental outcomes in the host. For example, IL-17 production by γδ T cells has been shown to aggravate autoimmune conditions (1, 2) and also be associated with immunosuppression that exacerbate tumour growth (3, 4). Such phenomena attributable to the varied cellular interactions of γδ T cells can be addressed by appropriate considerations of systems immunology and personalized approaches. In this regard, Chan et al. provided an updated account of crosstalk between γδ T cells and a variety of immune cells which collectively coordinates anti-tumor responses. Additionally, the cytokine milieu plays a major role in shaping the fate commitment of γδ T cells. Consistent with this, Song et al. discussed how cytokines and their combinations differentially direct the polarization of tissue-resident and tumour-infiltrating γδ T cells. Such experimental insights will inform strategies to manipulate the cytokine dependencies of γδ T cells to improve the cytotoxic function and *in vivo* persistence of administered T cells against tumors. Pei et al. elegantly showed that CD137 costimulation of Vγ9+Vδ2+ T cells using CD137L agonist diminished IL-10 receptor expression and alleviated exhaustion in these cells by mitigating the immunosuppressive tumor microenvironment (TME) mediated by IL-10. This increased Vγ9+Vδ2+ T cell efficacy against Epstein Barr virus (EBV)-transformed B lymphoma in a humanized mouse model. Hu et al. examined the immunosuppressive TME of hepatocellular carcinoma (HCC) which the authors showed close correlation with high expression of inhibitory checkpoint molecules, presence of tumor-promoting immune cells and various types of programmed cell death. This is associated with γδ T cell subtype imbalance characterized by selective depletion of cytotoxic Vδ2+ T cells and enrichment of Treg-like Vδ1+ T cells as well as poorer patient prognosis. These studies underscore the importance of performing greater in-depth analyses of the cellular networks and interplay of cytokines in the TME to accelerate the clinical translation of γδ T cell therapies.

Over the years, translating γδ T cell therapies from bench to bedside has encountered tremendous challenges. Saura-Esteller et al. expertly summarized the evolution of γδ T cell therapeutic strategies assessed in clinical trials that signifies past progress in γδ T cell research. With a growing list of companies developing γδ T cell-based or engaging therapies, the authors noted the high cost of manufacturing γδ T cell-based products due in part to requirement for multiple cytokines and prolonged *ex vivo* expansion process. Exemplifying continual efforts to reduce γδ T cell production cost, Ferry et al. described a one-step protocol utilizing a single cytokine to expand Vδ1+ T cells. In addition, the authors demonstrated that these cells were amenable to high efficiency transduction of chimeric antigen receptors (CARs). Going forward, development of other ways that harness the anti-tumor potency of γδ T cells, namely bispecific γδ T cell engagers and advanced γδ T cell genome-editing, is expected to widen the immuno-oncological applications of these cells.

## Author contributions

AT and AC discussed findings of various articles of the Research Topic in broader context of tumor immunotherapy. AT collated contributions and prepared final version of editorial by taking into account suggestions from AC and DK.

## Funding

This work is supported by the core funds of BTI, A*STAR and Industry Alignment Fund-Industry Collaboration Projects (IAF-ICP) grant ID I1801E0037 awarded to AH-MT. AMSC is supported by National Medical Research Council (NMRC) of Singapore-Centre Grant NMRC/CG21APR2002.

## Conflict of interest

The authors declare that the research was conducted in the absence of any commercial or financial relationships that could be construed as a potential conflict of interest.

## Publisher’s note

All claims expressed in this article are solely those of the authors and do not necessarily represent those of their affiliated organizations, or those of the publisher, the editors and the reviewers. Any product that may be evaluated in this article, or claim that may be made by its manufacturer, is not guaranteed or endorsed by the publisher.
